# Dynamics of social network structure for Alzheimer and Lymphoma scientific communities

**DOI:** 10.1186/s13062-015-0040-2

**Published:** 2015-02-20

**Authors:** Shahar Barbash

**Affiliations:** The Edmond & Lily Safra Center for Brain Sciences and The Department of Biological Chemistry, The Hebrew University of Jerusalem, Jerusalem, 91904 Israel

**Keywords:** Alzheimer’s disease, Lymphoma, Social network

## Abstract

**Abstract:**

It is generally assumed that sociology affects scientific progress but specific examples of this assumption are hard to find. We examined this hypothesis by comparing the social network structure and its dynamics over the last 16 years, for two common human diseases; Alzheimer’s disease, for which there has been very little therapeutic progress, and Lymphoma, were there has been significant therapeutic progress. We found that the Alzheimer’s research community is more interlinked (‘dense’) and more ‘cliquish’ than that of Lymphoma and suggest that this could affect its scientific progress.

**Reviewers:**

This article was reviewed by Vladimir Kuznetsov and Anthony Almudevar

## Findings

Scientific progress is affected by technologies, availability of funds and dominant hypotheses, but also by the social links between scientists. In other words, sociology affects scientific progress [1]. We examined this hypothesis by comparing two human diseases which seem to have different rates of progress: Alzheimer’s disease (AD), for which no substantial therapeutic development was made during the last decade [2], and Lymphoma, for which, at the same time, therapeutic treatment was significantly improved [3]. More specifically, we asked if there would be any identifiable changes in the social evolution of these two research fields during this time course. For that purpose, we built social networks for these diseases at four time points and compared the structural change over time. We focused on two, relatively easy to interpret, structural measurements: ‘node degree’ , which is a way to represent the tendency of a network to be ‘dense’ and ‘cluster coefficient’ which is an estimate of how ‘cliquish’ is a network. Tools and concepts for network analysis have developed substantially in recent years [4]. Although these tools were used to study network behavior of biological systems [5-7] and other varied topics, they have not been applied yet to study networks of scientific communities. We found the AD research community to be denser and more ‘cliquish’ then that of Lymphoma and suggest that these changes may affect scientific progress.

**Figure 1:**
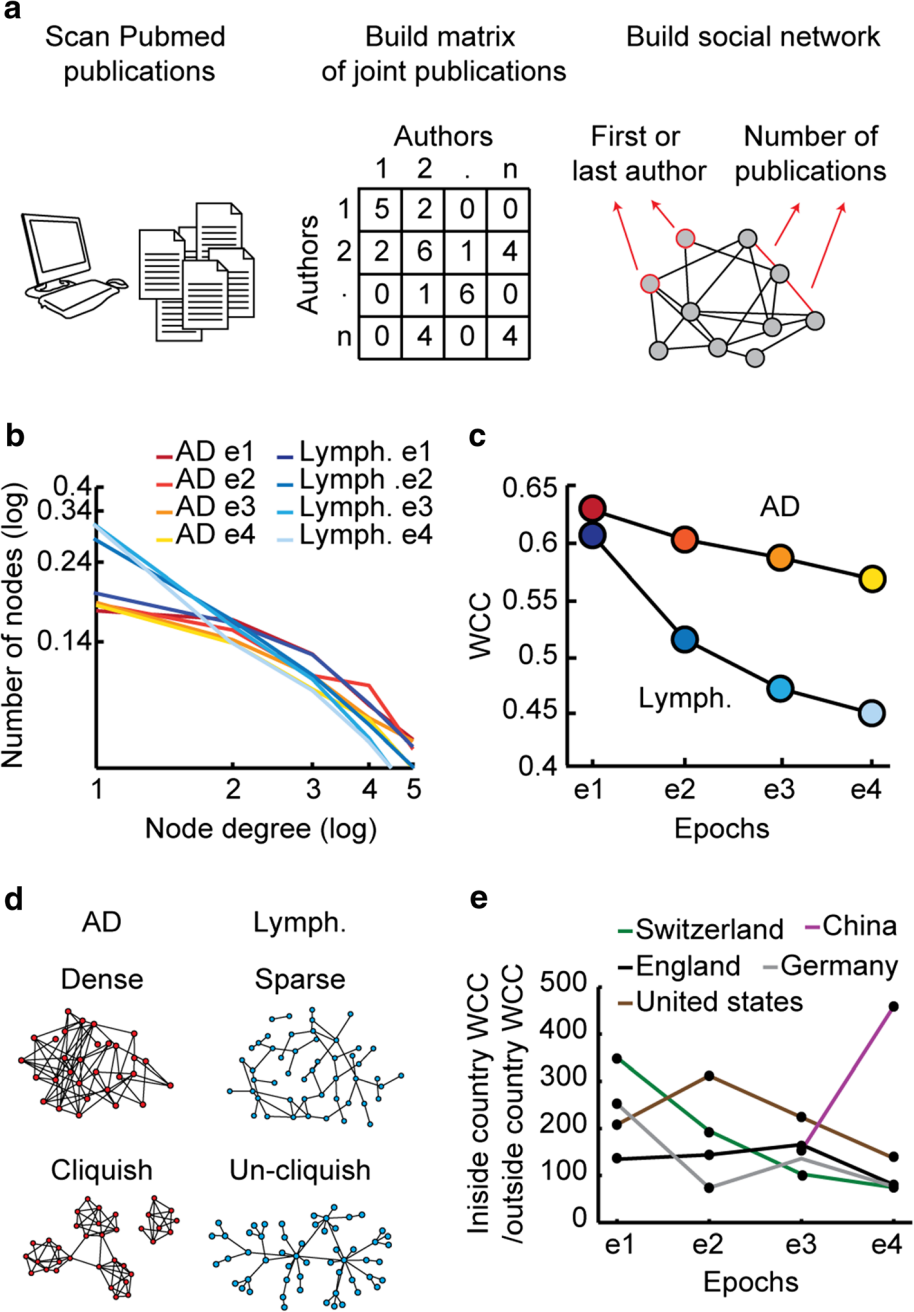
**Unique structure dynamics for AD and Lymphoma social networks.** **(a)** All PubMed publications of the last 16 ears, for each of the scientific field, were scanned. A list of either the last or the first author was prepared. Based on this list a connection matrix was built, with the number of joint publications for each researcher pair. Based on this connection matrix a neural network was built as explained in Horvath, Steve [4]. **(b)** For each network, the node degree distribution is plotted in a log scale. Note stable distribution for AD and an increase with time for Lymphoma (Lymph.). Kolmogorov-Smirnov (KS) test P value <0.05 for Lympohme epoch 1 and all other epochs and is not significant for any of the AD epoch comparisons. **(c)** For each network, the Weighted Cluster Coefficient (WCC) is plotted in time. KS test P value <0.05 between AD and Lymphoma WCC values. **(d)** AD social network is characterized by high density and cliquishness while the Lymphoma network is characterized by sparseness and low cliquishness. **(e)** Inside-country-WCC divided by the outside-country-WCC is plotted in time for different countries.

In order to build research-community-based social network we scanned the scientific literature represented in PubMed for publications with either the word ‘Alzheimer’ or ‘Lymphoma’ in the title or the abstract sections, over the course of the last 16 years (from 1998 to 2013). We have identified 64,439 publications dealing with AD and 110,331 dealing with Lymphoma. From these publications we extracted names of researchers that were either the first or the last authors. The middle authors were not included in order to exclude from the researches list authors that were only transiently involved in the specific scientific field and are not an integral part of it. Using these criteria, 25,715 Alzheimer’s researchers and 52,293 Lymphoma researchers were identified. Using this data we built connection matrices between researchers based on joint publications. A four year time window is an approximation of the time it takes for a researcher to be interested in a new concept and until he or she publishes an article on the subject. In addition, one has to have sufficient joint publications to be able to build a network. For these two reasons the connection matrices were collected separately for four epochs of four years each (1998–2001, 2002–2005, 2006–2009 and 2010–2013). One and two year epochs produced networks that are too spars to calculate the desired measures. Three year epochs showed a similar, although somewhat reduced effect as compared to four year epochs. If, for example, the first and second researchers have two joint publications in this epoch, the number 2 would be placed in the matrix beans (1,2) and (2,1). Next, we built the a weighted network, based on the principles introduced by Horvath and Dong [4], in which the nodes of the network are researchers and the links (edges) are the number of joint publication between researcher pairs. This analysis flow is described in Figure 1a and the produced connection matrices are available in the following link: https://drive.google.com/folderview?id=0B5KPcpJjNvdmcm9ST00ySnlCSnc&usp=sharing.

In order to measure density of the AD and Lymphoma networks we plotted the network degree distribution; a distribution (in log scale) of the total number of links each researcher in the network has, across the four epochs (Figure 1b). Distributions within the AD community were stable across the four time points while distributions within the Lymphoma community shifted from low number of researchers with small amount of links, in the first epoch, to large number of researchers with small amount of links, in the three last epochs. This means that the AD social network remained stably dense during the last 16 years, whereas the Lymphoma social network became sparser in the same time course.

We next examined the tendency of each of the social networks to include clusters of researchers. This network feature can be estimated by the Weighted Clustering Coefficient (WCC) which, when high, points at a ‘cliquish’ network. We calculated the WCC for each of the social networks, for each of the time epochs and plotted its dynamics in time (Figure 1c). Both the AD and the Lymphoma social networks showed a decrease in WCC with time. The WCC decreased faster in Lymphoma than in AD and although both networks started at the same level of WCC, at the end of the examined time period the AD WCC was considerably higher than that of Lymphoma. Therefore, the lymphoma social network became significantly less ‘cliquish’ than the AD network, during the last 16 years.

These results suggest a ‘dense’ and ‘cliquish’ structure for the AD network and a sparse and ‘un-cliquish’ structure for the Lymphoma network (Figure 1d).

We hypothesized that much of the clustering effect comes from inside countries and were interested in determining to what extent the different countries contribute to this effect. For that purpose we calculated, for each country, the inside-county-WCC divided by the outside-country-WCC, for each of the time epochs (Figure 1e). No difference was found between the two diseases and so they were examined jointly in this analysis. As expected, the inside-country WCC was always substantially higher than the outside-country-WCC (note that inside-county-WCC divided by the outside-country-WCC > > 1). Only countries in which there were at least 30 publishing researchers during the entire time period examined and at least 10 researchers in each epoch were considered. The numbers of publishing researchers in the different countries were Switzerland: 916, United States: 11,322, England: 4523, China: 336, Japan: 620 and Germany: 1067. A decreasing degree of inside-the-country clustering was observed in the last 16 years and 12 years in Switzerland and the United States, correspondingly. In contrast, during the last 8 years, China has shown increase in inside-the-country clustering. This shows that different countries have different contributions to the network clustering dynamics.

We have identified unique structure characteristics for the AD and Lymphoma research communities. During the last 16 years, the Lymphoma research community gradually became sparser and less cliquish, while the AD community retained stable and high levels of density and cliquishness. We chose to investigate two scientific fields in which there is a relatively wide consensus about one having significant scientific progress at both the basic and therapeutic levels and the other having only minor progress. If one would assume that network structural features affect scientific progress, one could attribute a ‘beneficial’ or ‘damaging’ effect for these structures. We found more sparseness and less cliquishness to be associated with better research progress (in the case of Lymphoma). Why should these two features be associated with better progress? One possibility is that a dense, cliquish network restricts entry of new concepts either due to the peer review process of scientific articles or the difficulty in finding fruitful collaborations; in other words, if introducing new scientific concepts places you outside the clique than the network would have a stable structure. The parameters that govern social network dynamics are numerous. We chose to discuss here only those that, based on our experience, are the ones with the highest potential to have a direct and major effect on the network.

Another possible assumption would be that some other factor (e.g. different funding, the nature of the disease) may affect both research progress and network structure. If this is the case we may interpret the cliquishness of the AD network, for example, as driven by the multifactorial nature of the disease. In this case one clique would study, for examples, epigenetics effects on AD and the other miRNA regulation. Another way to look at it would be to address the Lymphoma network as one big clique that work more or less with the same research hypothesis. Of course, both the ‘structure affects progress’ and ‘structure and progress are affected by another factor’ interpretations do not contradict each other and each may be part of the explanation.

The observed decrease in the tendency of inside-country clustering for Switzerland and the United States points to increasing degree of between-countries collaborations in researchers from these countries while the huge increase in this tendency for China points at the opposite. This indicates that the scientific communities in these countries undergo different structure dynamics and are probably shaped by different forces, such as policy and funding.

Taken together, these results show unique structure dynamics for two significant scientific communities, which allows speculation on the beneficial effect that each of these structures have on scientific progress. It will be interesting to see similar analyses for other scientific fields and disciplines in the future. Such analyses, which give a bird’s eye view of a scientific field and global comparison between fields, would be of great interest to network scientists, scientists working in the studied field, research institutes and policymakers alike.

## Reviewers’ comments

### Answers to comments of Referee 1, Vladimir Kuznetsov

Report form: The scientists, theoretically studying networks, believe that social network architecture and evolution analyses can help us to understand the structure and evolution of the real networks. Eventually, this helps to answer the question - how to allocate resources strategically and therefore boost the overall network efficiency, e.g., attract new investigators to join the scientific community working in a given research field, spawn new collaborations and receive funding, etc. ‘Big Data-driven’ approach is promising. The author of this work provides a statistical model of the interconnection of scientists linked to each other via joint publications. Specifically, their model is focused on networks of the scientists, whose names occurred in the first and last positions of PubMed-listed publications related to either Alzheimer’s disease or Lymphomas. The author analyzed the frequency distribution of the joint publications (the number of publication links within time interval) for the paired names. Based on their analysis, the author suggested that ‘Alzheimer’ disease study research community is ‘denser’ and more ‘cliquish’ than that of ‘Lymphoma’ and suggest that this could affect their scientific progress.

Major comments and criticism:

1. Original (raw) data and processed data used for model construction and analyses (e.g., matrix of joint publication) are not available. The reporting of original (raw) data is a key aspect of data-driven research study in context of its completeness, accuracy, reproducibility, and independent validation by peer review and the juxtaposition of research results, figures, statistical tables and author’s textual interpretation of their results. I assume that access/link to source data sets is a mandatory condition for the peer review process.
2. Missing data due to the link selection rule. Extraction of the linked names of researches is incomplete and biased, which could disproportionally change the links density and the structure of network.
  In his study, the author focused on comparison of the distribution functions, structure of the networks and network dynamics of the researchers in two scientific communities. Only researchers who were either the first or last authors of a paper listed in PubMed were extracted and defined as the links and network nodes. This publication link data was used for characterization of the node degree statistics of these two social groups of scientists and comparison of their network structures and dynamics. However, incorporating data from all corresponding authors and equally contributing first authors can increase the number of links and lead to more accurate and complete characterization of the empirical network model. If so, the values in the matrix of joint publications will be changed and lead to different network topology and probably interpretation of the analysis. I assume, that the author should take into account these comment and provide more accurate and robust network model.
3. False positive rare of the selection process should be estimated. The author used text mining procedure with either the word ‘Alzheimer’ or ‘Lymphoma’ in the title of the paper or the abstract sections. Such procedure may select some fraction of false-positive papers which actually did not focus of the study of ‘Alzheimer disease’ or ‘Lymphomas’. It should be important to estimate error rate of the selection criteria.
4. Processed data availability, statistics and statistical test results have to be reported. I expect also that the statistics table, numerical data, results of statistical tests supporting the figures and the main conclusions, should be available for the readers.
5. Uncertainty in the interpretation of main results ‘Social networks’ of scientific communities are multifactorial. However, the major ‘drivers’ (e.g. funding policy, disease complexity, distinct policy of the journal editor, social-economic driving factors, etc.) were not included in the studied model. Therefore, the interpretation of the results, based on analysis of the reported model could be less specific and conclusive. Alternatively, the influence of the driving factors onto the network model results can be evaluated via the model extension or/and its specific modification.
6. Collaboration network sample size. Based on visual inspection of the shape of the empirical histograms presented Figure 1b (left), the author claimed that the densities of compared networks are different.
  However, the degree of the difference is not quantified. In this context, it should be important to clarify the role of sample size in the comparison of the groups ‘Alzheimer’ and ‘Lymphoma’.
  The sample size is an essential parameter whose variation leads to re-shaping of the scale-dependent Pareto-like distributions (Kuznetsov VA, Genetics, 2002, 161:1321–32; Kuznetsov VA. Signal Processing, 2003 83: 889–910), which in the log-log plot could be approximated by a family of (skewed) positive curvature functions (e.g., Kolmogorov-Warring, generalized Pareto, gamma functions (Kuznetsov VA. 2003, Signal processing, 83: 889–910; Kuznetsov, VA. In: Computational and Statistical Methods to Genomics. (Eds. Zhang W, Shmulevich I.) Springer, USA, 2nd edition, 2006: 160–208). According to my visual inspection of the shape of the histograms shown on Figure 1b, the histograms could be better approximated by the scale-dependent frequency distribution model(s). If so, the scale-free network distribution approach should be not applicable and a role of sample size should be carefully investigated and discussed.
7. Temporal evolution: binning intervals (into 4 year- epochs) were not optimized and statistically supported. Statistical preference of the 4-year cut-offs vs alternative (e.g., 1, 2, 3 year cut-offs) should be reported. It could be important to identify a more appropriate mathematical model of the node degree statistics. This is because the shape of the empirical skewed histogram and the node degrees can be quite sensitive to the number of binning intervals and distribution of binning cut-off values.
  It is also possible that reduction of sample size due to the splitting of the initial data could lead to artificial reduction of the network structures (modules) and its connectivity and network diversity. It should be important to construct and analyze the histograms for each group (‘Alzheimer’ and ‘Lymphoma’) without splitting based on the time interval (epoch) subsets, and to compare the robustness of network characteristics between these entire groups and the subgroups selected for different time binning intervals.
8. The comparative analysis of the frequency distributions and classification of the network structures is incomplete.
  The classification of the network structures (modules) for each studied research community should be reported and compared using appropriate statistical tests Term ‘clique’ should be defined mathematically. The statistics of the ‘cliques’, identified in the distinct and similar social groups, should be reported. Other basic network properties such as the centrality measure, vertex similarity/dissimilarity, fraction of single links and dynamics of these features should be characterized (Newman M.E.J., 2012, Nature Physics, 8:25–31).
9. Figure 1d showed selected examples of sub-networks, but it should be not enough. How these sub-networks are representative in the studied groups? The author should demonstrate all structures and provide the statistical analysis of these structures for studied groups.
  Quality of written English: Acceptable

## Author rebuttal

1. Following is a link with the 8 publication matrices as mat files. This link was also incorporated into the revised version in the https://drive.google.com/folderview?id=0B5KPcpJjNvdmcm9ST00ySnlCSnc&usp=sharing.
2. Defining only the first or last authors as the network edges arise from the fact that both Lymphoma and Alzheimer’s research fields are extremely active and a huge number of labs and researchers are involved in them. Looking at the population of authors/researchers, the ‘middle’ authors very rarely show up in more than one publication in the same field. Their presence in the scientific field is sporadic and not stable. Therefore, their exclusion from the network actually decreases the noise level and increases robustness. These considerations for building network are described in the main text. Upon inclusion of the middle authors not only that the values of joint publications (links) would have been different but the whole network would have been different; number of edges would have been increased in about 10 fold and the network would be extremely sparse, because of generally small involvement of the ‘middle’ authors in the scientific field. Hence, stable edges (first and last authors) were taken into consideration while unstable, transient edges (middle authors) were not. Regarding equally contributing first authors – these cases are less than 5% form the examined articles and hence would have a negligible effect.
3. When setting the criteria for Alzheimer’s articles, I have checked for false positive rate: among 20 articles with the word Alzheimer in their title or abstract, only one article was unambiguously not about Alzheimer. The rest were about varying aspects of the disease, at least partially.
4. I added the corresponding Kolmogorov-Smirnov test P values in the legends for Figure 1b and c. This test helps determining if two empirical distributions were drawn from the same population distribution or from two different ones. Note that each value in these graphs was measured for a single network and so do not have variance, so ANOVA is not practical here.
5. I agree that the multifactorial nature of the network makes the interpretation hard; this is why the discussion is of inconclusive nature and suggests several potential interpretations for the observed effect. At any event, I emphasized even more the multifactorial nature of the network (page 5, bottom). I am not sure what did the reviewer meant by “evaluation via the model extension or/and its specific modification”. This comment might be linked to an option I considered while performing this study; to perform historical screen of major scientific events that are relevant to the examined fields, such as drug or budget approvals. The conclusion I came to is that first, it is almost impossible to decide which event should be included and which should be excluded from such a screen, and second, and more importantly, the time resolution of the network would not allow any serious comparison between the network dynamics and the historical screen. This is because the 4 year epoch for each network is a minimum to build a network that is meaningful scientifically (taking into account the time it takes for an article to be published, see more about that in a comment below).
6. Quantifying the degree of the change was addressed in a previous comment about statistical tests. I do not believe that modeling differently the network distribution and going into approximation considerations would contribute to the main argument in this manuscript. The main argument is based on the difference between distributions and was shown to be significant. Whether each of the distribution could be fit with a specific function, although interesting and important by itself, is outside the scope of this article.
7. The rationale behind the 4 year epochs is mentioned in page 3, bottom, but perhaps not detailed enough. Optimization actually was performed; 1 or 2 year epochs produce not enough joint publications, in these time epochs the reported effects were not seen. We attribute this to the extreme sparseness of these networks, the 3 year epoch showed a similar effect albeit somewhat reduced compared to the 4-year epoch. That led us to use 4-year epochs which is also reasonable based on our experience. I have emphasized this rationale in the corresponding place.
8. ‘Cliquishness’ is the term used to describe clustering in a social network. It was calculated as in Hovarth’s “Geometric interpretation of gene coexpression network analysis”, as mentioned in the manuscript. Other properties, some of them are the ones mentioned by the reviewer, did not show an effect between the two scientific fields. Also, besides the “centrality” measure, we could not find any intuitive interpretation to these network characteristics for a social network, and hence decided not to report them. These considerations are also mentioned in the manuscript (page 2, bottom).
9. The difference in sparseness and cliquishness, although significant, is not global enough and strong enough to be seen visually when looking at the two networks. Nonetheless, measuring these characteristics show an effect. This is often the case when the difference between two networks is a subtle, averaged effect. The purpose of Figure 1d is to ease on the non-specialist reader and make the network measures intuitive.

### Answers to comments of Referee 2, Anthony Almudevar

Summary

The authors construct a research social network, based on published manuscript co-authorships, for Alzheimer Disease and Lymphoma research, for four consecutive 4 year periods. Differences in these networks (they may be more or less ‘cliquish’) are offered as explanations for the greater progress in Lymphoma research.

Major comments

1. The authors introduce the term ‘scale-free’ essentially as a synonym for ‘uncliquish’ structure (pages 4,8). The formal definition for a ‘scale-free’ network is not given in the paper. My understanding is that a ‘scale-free’ network is one in which the degree distribution follows a power-law [ P(k) \propto k^{−d}, d > 0 ]. In this case the plots in Figure 1b would be approximately linear, which seems to be the case for the e2,e3 and e4 Lymphoma networks. However, I don’t believe that the idea that the ‘scale-free’ property and the ‘uncliquish’ property are necessarily associated is supported by the literature.
2. The ‘within-country’ analysis (pages 4–5) is rather imprecise.
3. The authors state that Lymphoma research has been generally more successful (greater ‘therapeutic process’ , page 2) than for Alzheimer’s disease. The open question is whether or not some of the explanation for this can be found in the differences in social network structure. Possibly, the greater ‘cliquishness’ of the Alzheimer’s social network hampers progress. Of course, there must be a very large number of metrics of many kinds that would also differ between the two groups (that one is cancer and the other a neurological disease might account for many of them), and there is really nothing in the paper which establishes any sort of explanatory relationship.

Minor comments

- page 2 “… specific examples [of] this …”
- page 3 “… that were either the first [or] the last …”
- page 3 There is a second author (J. Dong) in “Horvath, Steve [4]”
- page 4 The plot does not seem to be a histogram
- page 6 “… allow a bird’s [eye] view … “or” … allow a bird’s [] perspective …”

4. Are the networks in Figure 1d actually networks constructed from the data, or simply examples of graphs with the stated property?
5. Quality of written English:Not suitable for publication unless extensively edited.

## Author rebuttal

1. I humbly agree. This inaccuracy was changed.
2. The analysis compares between the clustering coefficient of one sub-network and another. I find it logical.
3. I read this comment, similar the first reviewer’s comment, as a comment about the inconclusive nature of the discussion. As was my respond to the first reviewer, I basically agree with this critic and so incorporated it into the original discussion. Nonetheless, I have also incorporated remarks about explanatory relationship that are based on my experience as a researcher.
4. I thank the reviewer for these corrections. They were all incorporated into the revised manuscript.
5. These are examples of graphs with the stated property. See answer to first reviewer’s comment.
6. Extensive editing was made.

## Second round of reviewer’s comments

**Reviewer 1:** Q5-Q6. For comparison analysis of the skewed (Pareto-like) distributions presented on Figure 1b the sample sizes can be essential issue. The sample size (the total number of the subjects (or network nodes)) is important parameter of such discrete distribution functions, whose value variation leads to re-shaping of the distribution function. It is well-defined property of the scale-depended network distributions [Pareto-like distributions (Kuznetsov VA, Genetics, 2002, 161:1321–32; Kuznetsov VA. Signal Processing, 2003 83: 889–910). In this case the empirical frequency distributions taken at random from the same population, but forming different sample size statistics become significantly different when difference between given sample sizes increases. If so, author’ claim “the main argument is based on the difference between distributions and was shown to be significant” should be proved using an appropriate sampling and statistical testing (may be not only by visual inspection of the functions on the plots).

Q9. The author should be report that “the difference in sparseness and cliquishness, although significant (how much? VK), is not global enough and strong enough to be seen visually when looking at the two networks. Nonetheless, measuring these characteristics show an effect”.

**Author:** The statistical tests are reported in the figure legend.

**Reviewer 1:** Is that effect statistically significant, how much? Statistical properties of the networks structures in the studied groups should be supported.

**Author:** statistical properties are reported at the top of page 3.

**Reviewer 1:** “The purpose of Figure 1d is to ease on the non-specialist reader and make the network measures intuitive”. The quantitative network analysis oriented also on the specialist readers should be useful.

**Author:** I believe this figure serves both kinds of readers.

## Abbreviations

AD: Alzheimer’s disease
WCC: Weighted clustering coefficient
